# Cell Membrane-Based Biomimetic Nanoparticles and the Immune System: Immunomodulatory Interactions to Therapeutic Applications

**DOI:** 10.3389/fbioe.2020.00627

**Published:** 2020-06-17

**Authors:** Manuela Sushnitha, Michael Evangelopoulos, Ennio Tasciotti, Francesca Taraballi

**Affiliations:** ^1^Department of Bioengineering, Rice University, Houston, TX, United States; ^2^Center for Musculoskeletal Regeneration, Houston Methodist Research Institute, Houston, TX, United States; ^3^Orthopedics and Sports Medicine, Houston Methodist Hospital, Houston, TX, United States

**Keywords:** biomimetic nanoparticles, immune system, nano-bio interface, immunomodulation, drug delivery

## Abstract

Nanoparticle-based drug delivery systems have been synthesized from a wide array of materials. The therapeutic success of these platforms hinges upon their ability to favorably interact with the biological environment (both systemically and locally) and recognize the diseased target tissue. The immune system, composed of a highly coordinated organization of cells trained to recognize foreign bodies, represents a key mediator of these interactions. Although components of this system may act as a barrier to nanoparticle (NP) delivery, the immune system can also be exploited to target and trigger signaling cues that facilitate the therapeutic response stemming from systemic administration of NPs. The nano-bio interface represents the key facilitator of this communication exchange, where the surface properties of NPs govern their *in vivo* fate. Cell membrane-based biomimetic nanoparticles have emerged as one approach to achieve targeted drug delivery by actively engaging and communicating with the biological milieu. In this review, we will highlight the relationship between these biomimetic nanoparticles and the immune system, emphasizing the role of tuning the nano-bio interface in the immunomodulation of diseases. We will also discuss the therapeutic applications of this approach with biomimetic nanoparticles, focusing on specific diseases ranging from cancer to infectious diseases. Lastly, we will provide a critical evaluation on the current state of this field of cell membrane-based biomimetic nanoparticles and its future directions in immune-based therapy.

## Introduction

The ultimate goal of nanoparticle-based drug delivery is to achieve the therapeutic accumulation of a given treatment to the site of disease while minimizing off-target effects. This requires the use of materials that act as drug delivery vehicles to carry small molecules or biologics to the target site. A host of materials, including both organic and inorganic, have been tested to date. Given the extensive variety of biomaterials that can be used as the building blocks for the synthesis of these nanoparticles (NPs), it raises the question of which criteria and design principles are critical when selecting the ideal material (Yu et al., 2016). The success and limitations of tested materials have revealed three essential tasks that NPs must accomplish to achieve their drug delivery objective. First, NPs must have an appropriate circulation time that enables them to reach the target site (Yoo et al., 2010). Next, these NPs must be capable of only acting upon disease tissue while leaving healthy tissues intact (Moghimi et al., 2001; Friedman et al., 2013). Lastly, NPs must be composed of a biodegradable material that can be cleared from the body without negative effects (Naahidi et al., 2013). At the heart of these criteria is the underlying need for the chosen NPs to engage with the complex biological environment of the human body. In particular, the immune system plays a crucial role in mediating the biological interactions that directly affect the success of the chosen NP to achieve the previously listed tasks.

In fact, the human body possesses a highly specialized system for sustaining homeostasis: the immune system. The immune system is vital for not only protecting the body from harmful pathogens and foreign materials, but also in the identification of abnormalities within cells and tissues (Chaplin, 2010). The role of the immune system can be viewed as a two-sided coin. On one side, introduction of a biomaterial *in vivo* through systemic administration instigates an immune response to clear the foreign material from the body (Zolnik et al., 2010). This clearance impedes the therapeutic efficacy of NPs, either due to their inability to reach the target site or the neutralizing effects of immune cells that prevent them from acting upon the diseased tissue. On the other hand, the immune system is fundamental to the pathophysiology of disease manifestation. In fact, many of the diseases that NPs target present inflammation, an immune response that aids in the recruitment of immune cells to the disease site (Chen et al., 2018). The presence of this inflammation results in the overexpression of receptors or release of cytokines, molecular features that can serve as targeting mechanisms that bring the NPs to the disease site.

Given the key role that immune cells play in regulating their therapeutic efficacy, NPs must be capable of engaging directly with the biological components of the immune microenvironment. On the cellular level, NPs are capable of communicating with the immune system through their surface features. This communication between NPs and immune cells is mediated by the interactions at the nano-bio interface, which refers to the region where the nanoparticle surface comes in direct contact with its surrounding biological environment (Nel et al., 2009). This process is particularly critical during circulation as the NP surface is the first component an immune cell interacts with. The subsequent series of interactions that occur at this nano-bio interface involves both direct and indirect signaling cues that determine how the immune cell will respond to their presence in the bloodstream. Therefore, the composition and physicochemical features of the NP surface greatly determine how they are perceived by the immune system and, thereby, can regulate their ability to overcome the biological barriers posed by the immune system (Wang and Wang, 2014; Liu and Tang, 2017).

While previous approaches in nanomedicine aimed to minimize the immune interactions with NPs (i.e., “biologically inert systems”), recent years have seen a burgeoning interest in the field of biomimetic NPs, particularly cell membrane-based NPs. This emerging class of drug delivery vehicles capitalizes on the natural interactions between NPs and the biological components of the human body while mimicking the features and functions of native cells (Parodi et al., 2017). Thus far, a host of novel biomimetic technologies have been developed. These NP formulations have used a combination of whole cells (Evangelopoulos et al., 2020), cell ghosts (Toledano Furman et al., 2013), and the incorporation of cell-derived membrane proteins to mimic the biological characteristics and functions of native cells, enabling them to evade immune clearance and increase therapeutic efficacy (Liu et al., 2019). These platforms have demonstrated the potential of using biomimicry as a means to overcome the biological barriers posed by the immune system, with a specific emphasis on minimizing their clearance from the body prior to reaching their intended target (Perera and Coppens, 2019). Furthermore, this biomimetic approach enables NPs to communicate directly with immune cells by presenting transplanted cellular components and signaling cues to favorably modulate the immune response inherent within the disease site (Dacoba et al., 2017).

This review will provide critical insights and key perspectives on the current state of the field of immunomodulatory cell membrane-based NPs. We will begin by describing the relationship between NPs and the immune system, highlighting how the latter can serve as both a barrier and a target for these drug delivery systems. We will then highlight the role of the nano-bio interface in the ability of NPs to communicate with the biological environment in the body. Next, we will describe the recent emergence of biomimetic nanoparticles and explore methods used to mediate immunomodulation in diseases ranging from cancer, to cardiovascular disease to infectious diseases, emphasizing novel technologies that capitalize on the interactions occurring at the nano-bio interface. Finally, we will provide an analysis on the future directions of this growing research field and ways to address the current challenges faced in the clinical translation of biomimetic NPs.

## Role of the Immune System: A Two-Sided Coin

In order to dissect and analyze the relationship between NPs and the immune system, the topics discussed in this review will focus on two aspects of this relationship. On one hand, immune-mediated clearance mechanisms will be presented as the primary barrier to NPs targeted delivery. On the other hand, the prevalence of inflammation across many disease conditions and NPs interactions with immune cells at the diseased site will be highlighted as potential targets for immunomodulatory behavior of these NPs.

### The Immune System as a Barrier

Given its key function of recognizing and eliminating foreign bodies, the immune system hinders the localization of NPs to the site of disease. Upon injection into the bloodstream, NPs are quickly removed from circulation through two main routes: mononuclear phagocyte system (MPS), and natural clearance by filtering organs (Blanco et al., 2015). While the latter mechanism is directed by the size of the particles, the former involves direct communication between NPs and immune cells. Phagocytosis of foreign bodies, a process mediated by components of the immune system, is one of the mechanisms by which NPs are cleared from the body (Gustafson et al., 2015). Mediators of this process include opsonin proteins and monocytes. Circulating opsonin proteins bind to the surface of NPs, marking them for macrophage uptake (OwensIII, and Peppas, 2006). Once marked, NPs trafficked to the primary MPS organs are eliminated by the macrophages present in these organs. These include red pulp macrophages found in the spleen, Kupffer cells found in the liver and alveolar macrophages found in the lungs (Hume et al., 2019). As a result, this uptake of NPs from the bloodstream results in non-specific distribution, with greater accumulation in the liver and spleen (Song et al., 2014; Blanco et al., 2015). In fact, biodistribution studies on commonly used NPs have corroborated that these organs do indeed show higher concentrations present within them (Alexis et al., 2008; Cataldi et al., 2017; Feng et al., 2018). These immune-regulated clearance mechanisms thwart NP delivery to the diseased tissue and, thereby, limit their potential therapeutic impact. It should be noted that there are circumstances in which the natural NPs targeting of phagocytic cells and accumulation in MPS organs has been exploited. In fact, multiple studies have shown how this strategy can be been leveraged for therapeutic applications (Bartneck et al., 2014; Cui et al., 2019; Evangelopoulos et al., 2020). However, the focus of the discussion in this review paper will remain on how the immune system can prevent preferential accumulation in other target organs.

Therefore, recent efforts in nanomedicine have aimed to improve the ability of NPs to overcome this barrier posed by the immune system. In particular, researchers have focused on the design of materials that enable NPs to evade immune recognition by presenting surface properties that prevent them from being marked as foreign. This strategy has come in the form of non-fouling coatings that prevent the attachment of opsonin proteins or integration of self-marker proteins found on native cells (Schlenoff, 2014; Sosale et al., 2015). By displaying these features to MPS-specific circulating immune cells, NPs can circumvent the issue of immune-mediated clearance by strategically communicating messages at the nano-bio interface. As a result, these NPs possess a greater ability to reach the target disease site and, thereby, have a greater therapeutic efficacy *in vivo*.

### The Immune System as a Target

Although the immune system can be viewed as a key biological barrier NPs must overcome on their journey from the injection site to the disease site, it can also be an opportunistic target for these drug delivery vehicles. Given that the therapeutic efficacy of these NPs hinges on their ability to selectively target the disease tissue, researchers have relied heavily on active targeting mechanisms to achieve this goal. Components of the immune system, especially in the disease context, represent targets that NPs can exploit in order to increase their accumulation in a specific site. This targeting strategy takes on two forms: (i) design of NPs to target the inflammation present across various diseases or (ii) enable NPs to directly communicate with the immune cells present in the local microenvironment.

Inflammation, the coordinated biological response to pathogens or damaged cells, is a characteristic feature across many diseases, ranging from cancer to infections (Rock and Kono, 2008). This immune response can be characterized as either short-term or long-term. Although short-term inflammation results in healing, chronic inflammation is a dysregulated and maladaptive response that involves active inflammation, cellular breakdown, and unsuccessful attempts at repair (Chen et al., 2018). The latter is the form seen in most diseases such as cancer, atherosclerosis, and autoimmune diseases (Coussens and Werb, 2002; Lopez-Candales et al., 2017; Duan et al., 2019). A host of cellular and molecular pathways are involved in the inflammatory response, with variations in the proteins involved stemming from the underlying disease. Generally, inflammation is marked by high levels of chemokines and cytokines which serve as attractants for cytotoxic molecule-producing leukocytes (Feghali and Wright, 1997). The prevalence of this inflammatory state across many disease conditions allows for it to serve as a target for NPs can exploit for preferential accumulation. This can be achieved by NPs binding to overexpressed receptors on inflamed tissue or through detection of the cytokines present in the local environment (Jin et al., 2018). Therefore, the molecular features of inflammation are means by which NPs can home to the target site.

In addition to targeting the molecular features of inflammation, NPs can also communicate directly with the immune cells present in the local microenvironment. As previously discussed, the primary function of the immune system is the maintenance of homeostasis. When this state of balance is altered during the progression of a disease, the immune system quickly responds by recruiting specific populations of immune cells to respond and restore the local environment to equilibrium (Kotas and Medzhitov, 2015). The balance of when and which immune cells arrive to the disease site is crucial in coordinating a proper response. In order to achieve this communication and modulation of immune cells, NPs can serve as artificial antigen presenting cells (APCs). These engineered NPs mimic the natural interactions between dendritic cells (DCs) and *T* cells. In particular, these artificial APCs possess the peptide MHC complexes needed for *T* cell receptor specificity and co-stimulatory molecules that initiate activation of *T* cells (Wang et al., 2017). Lastly, the NPs can also be loaded with cytokines to supplement *T* cell expansion induced by their activation (Eggermont et al., 2014). Therefore, these NPs act as the DCs that would normally interact and engage with *T* cells in the disease context. The utility of this strategy has been demonstrated by several NP platforms including iron oxide NPs and liposomes conjugated with the previously discussed ligands, validating the ability of these NPs to alter the immune cell population (Prakken et al., 2000; Hickey et al., 2017). Overactivation of the immune system can also prove to be detrimental and may induce further damage to the disease site. This is especially the case in autoimmune disorders where the body’s immune system attacks its own cells and warrants the modulation toward a reduced response. In fact, this inflammatory state is orchestrated by the interactions between APCs and *T* cells (Mackern-Oberti et al., 2015). Once again, NPs can serve as artificial APCs as previously described. However, these NPs target specific antigen receptors on T cells via self-peptide-MHC complexes to induce tolerance (Probst et al., 2014). As a result, these NPs now behave much like the tolerogenic DCs that can stimulate regulatory *T* cells while suppressing cytotoxic *T* cells in order to modulate the overactive immune response (Steinman et al., 2003; Serra and Santamaria, 2015). Examples of these immunomodulatory NPs include carbon nanotubes to promote lung immunosuppression and polymeric NPs to induce regulatory DCs (Tkach et al., 2011; Maldonado et al., 2015). Taken together, NPs can mediate and modulate the immune response by communicating with specific immune cells vital for mounting the appropriate response for a given disease.

## Role of the Nano-Bio Interface

The nano-bio interface is comprised of a complex and dynamic environment in which the NP surface actively engages with the biological components of the surrounding system. The interactions that occur at this surface are crucial in determining the *in vivo* fate of NPs. A NP’s physicochemical properties, which include size, surface charge, and functionalization, actively contribute to the exchanges that occur here (Nel et al., 2009). In fact, one can view these characteristics as the language that NPs use to communicate with the cells they come in contact with. This communication is determined by both what the nanoparticle sees and what components of the biological environment see on the NP’s surface. The nano-bio interface interactions with the immune system consists of two arms – the exchanges that occur while the particles are in systemic circulation and those that occur in relation to the target tissue.

Upon entry into the bloodstream, NPs face a complex and dynamic environment of cells and proteins that immediately begin interacting at the surface interface. Physicochemical properties such as size, geometry and surface charge play significant roles on the stability of the NPs while in circulation. For example, NPs that are roughly in 100 nm in size have demonstrated longer half-lives in the blood, while discoidal-shaped particles exhibit improved margination to the vessel walls when compared to their spherical counterpart (Alexis et al., 2008; Gentile et al., 2008). Taken together, both of these NP features improve the NP’s ability to avoid phagocytosis/clearance and interact with the endothelium. By enhancing this ability, we can increase the probability of the NP to extravasate out of circulation and reach the target tissue. Furthermore, neutral and negatively charged particles reduce adsorption of serum proteins (i.e., albumin, opsonins) onto the surface (Yamamoto et al., 2001; Aramesh et al., 2015). As previously discussed, the MPS plays a significant role in determining the behavior and outcome of NPs following systemic injection. As opsonin proteins coat their exterior, NPs undergo significant changes in their surface composition, which in turn affects their interactions with other cells (Xiao and Gao, 2018). The formation of this protein corona has been shown to mediate the interactions occurring at the nano-bio interface. From the perspective of a circulating macrophage, the presence of the opsonin protein on the NP surface communicates a message of the presence of a foreign body that must be immediately cleared. In contrast, a NP with a polymer coating or negative surface charge can minimize the binding of opsonin proteins, enabling the NP to continue its journey to the target site with reduced uptake by circulating cells that will hasten its clearance from the body. In fact, researchers have relied heavily on the former as a means to minimize cell-to-particle interactions in the bloodstream. Commonly used surface functionalization techniques to address this issue have included coatings with poly(ethylene glycol; PEG), chitons, dextrans, and other polymers (Gref et al., 1994; Mitra et al., 2001; Jokerst et al., 2011). In contrast to traditional chemical coatings, others have also utilized the integration of “self-marker” proteins, such as CD47 and CD45, as a means for NPs to communicate a message of “don’t eat me” to circulating monocytes (Rodriguez et al., 2013).

Scavenger receptors represent another key class of molecules that determine the interactions between NPs and the cells that they encounter *in vivo*. These receptors (e.g., SR-B1, CD36, and MARCO) are known to be expressed on many cell types, including the macrophages and endothelial cells that NPs interact with while in the bloodstream and at the target tissue (Shannahan et al., 2015). Binding to these receptors results in cellular uptake, which has been shown to be both beneficial and detrimental to NP interactions at the nano-bio interface. For example, high levels of SR-B1 on tumor cells has been exploited to improve targeting of NPs to ovarian and colorectal cancer (Shahzad et al., 2011). On the other hand, macrophage uptake of silver NPs was found to be mediated by SR-B1 while inducing increased expression of proinflammatory cytokines (Aldossari et al., 2015). Although the expression of ligands on the NP surface for scavenger receptors enabled accumulation to target sites, the same receptors also resulted in unfavorable uptake by macrophages which reduced the circulation times of these NPs. These examples highlight how the nano-bio interactions can have both positive and negative outcomes for the NPs. Therefore, tuning and balancing of these interactions at the interface is vital for the successful therapeutic applications of NPs.

Beyond overcoming the barriers encountered in the bloodstream, NPs must also be designed to communicate and stimulate therapeutic responses via interactions with immune cells involved in disease progression. This communication is mediated by the messages a NP communicates through its surface features. This is due to immune cell activation being largely stimulated by the presence of antigens on cell surfaces or other molecules an immune cell senses and feels in its biological environment (Chaplin, 2010). Therefore, NPs can serve as artificial APCs that express surface features that can either activate immune cells or modulate the expression of pro- or anti-inflammatory genes that stimulate the infiltration of specific subpopulations of immune cells while thwarting the proliferation of others (Hickey et al., 2017). This ability of NPs to tune immune cells begins with the shape of the NP itself. To mimic native APCs (i.e., DCs) that are not spherical in shape, NPs with an ellipsoid and nanotube morphology have been shown to better engage with the target immune cell (Fadel et al., 2008; Sunshine et al., 2014). By increasing the surface area of contact, these NPs improve their ability to be seen by *T* cells and mediate key ligand-receptor interactions (Eggermont et al., 2014). As previously discussed, the integration of stimulatory and regulatory molecules on the NP surfaces facilitates communications to immune cells. For example, as *T* cells bind to specific NP moieties (e.g., MHC peptides, CD80, and CD86), they can be stimulated to expand cytotoxic *T* cells that infiltrate the tumor or increase the regulatory *T* cell population to downregulate the overactive immune response underlying an autoimmune disorder (Kim et al., 2004; Rhodes and Green, 2018). Therefore, by expressing the molecules found on native immune cells on their surfaces, these NPs communicate messages directly to the interacting immune cell (e.g., DC). Seeing these molecules, this immune cell goes on to trigger a subsequent cascade of events that primes the immune response to the disease in a therapeutically favorable manner. This immunomodulatory behavior can prove to be powerful for the therapeutic response of injected NPs, especially when this response is triggered simply by the interactions occurring at the nano-bio interface.

## Emergence of Biomimetic Nanoparticles

**FIGURE 1:**
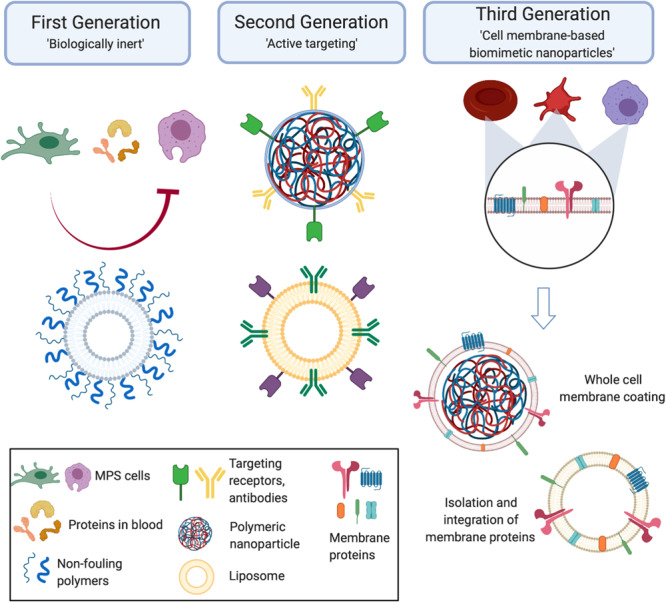
Generations of nanoparticles. Early generations of particles were biologically inert and used non-fouling coatings to prevent nanoparticles from interacting with the cells they encounter *in vivo*. From here, the next generation of nanoparticles became active targeting molecules that enabled the nanoparticles to reach the disease site and engage with the local environment. Taking inspiration from nature, the third generation of cell membrane-based biomimetic nanoparticles mimic the surface features of native cells by utilizing whole cell membrane or membrane protein functionalization onto synthetic carriers. Created with Biorender.

Over the course of the past several decades, the field of nanomedicine has seen the development of several generations of NPs. With each generation, researchers have made large strides in improving the therapeutic efficacy of these platforms. This progress has been reinforced by the work across many disciplines that have provided valuable insight in the biology behind the diseases and the interplay between synthesized materials and their behavior *in vivo*. We see this in the evolution of the generations of NPs. Initially, the emphasis was placed on the development of NPs that were “biologically inert” (Figure 1). This stemmed from the goal of reducing the interactions between a NP and the immune cells that had been proven to be detrimental to the *in vivo* fate of the particles, which includes macrophages and other components of the MPS (Qie et al., 2016). Over time, researchers recognized that this strategy also was not sufficient in successful delivery of a payload to a target site. In fact, NPs had to not only minimize surface interactions with some cell types, such as those with components of the MPS, but also actively engage with the cells present in the microenvironment of the disease. This led to the rise of “targeted NPs” that were surface functionalized with molecular signatures (i.e., peptides, antibodies) that enabled them to specifically reach and communicate with target tissues (Sapsford et al., 2013; Figure 1). Although this strategy did result in improvements in the specificity of NP accumulation, it also had its limitations. In particular, functionalization with one or a few markers was insufficient, particularly in engaging with the complex communication occurring at the nano-bio interface (Crist et al., 2013). In the search for a more comprehensive solution for this challenge, researchers looked to native cells as a source of inspiration. From here came the emergence of biomimetic NPs. This third generation of NPs aims to develop particles that recapitulate the features and surface characteristics of native cells using a more *in toto* approach (Luk and Zhang, 2015; Parodi et al., 2017; Figure 1). As these NPs act and behave much like the body’s own cells, biomimetic NPs can facilitate the interactions and communications at the nano-bio interface with greater ease.

During the early years of the first generation of NPs, these NPs were synthesized with one primary intention – utilize the natural transport phenomena occurring in the body to passively carry NPs from the injection site to the disease site, while minimizing interactions with the biological components of the body. The synthesis of this first generation of NPs centered on testing different chemical compositions, size and non-fouling coatings (Faraji and Wipf, 2009; Albanese et al., 2012). However, it became apparent that the development of NPs that are completely agnostic to the *in vivo* environment was impossible. Therefore, the second generation of NPs saw a shift toward more targeted, bioactive carriers. Specifically, these drug delivery vehicles were designed to enable them to reach the target disease and reduce non-specific biodistribution (Mout et al., 2012). Commonly used methods included the attachment of affinity ligands, such as antibodies, peptides and small molecules (Friedman et al., 2013). This growing trend in surface functionalization represented early attempts at directing active communication between a particle and surrounding cells at the nano-bio interface. In contrast to the first generation of NPs, this subsequent generation comprised of particles encoded with messages on their surface that enabled them to mediate interactions with other cells. In the context of communication with immune cells, this strategy has taken on two forms that address the two-sides of the coin discussed previously. On one side, NPs were functionalized with markers that reduced their uptake and clearance by the MPS (Zhou and Dai, 2018). On the other hand, studies have also shown how the integration of affinity ligands enables NPs to selectively reach the target site and engage with the immune cells present in that microenvironment (Chen et al., 2012; Schmid et al., 2017). Although the use of these molecules has demonstrated promising results, attachment of these molecules as single moieties in their non-native form can inhibit their full function. In fact, the conjugation chemistries used to attach these molecules can result in variations in their orientation and densities on the NP surface, resulting in a change of function or a complete loss of function (Rambukwella et al., 2018; Alkilany et al., 2019).

Given the shortcomings of the second generation of active targeting NPs, researchers looked to nature as a source of inspiration in developing NP formulations for specific applications. Herein, we have seen the emergence of biomimetic NPs, the third generation of NPs who mimic the features of nature to enhance their therapeutic effects *in vivo*. The biomimicry achieved by these NPs can be through chemical, physical, or biological means. For example, calcium phosphate NPs mimic the structural and compositional similarity of native bones and teeth (Kalidoss et al., 2019). As a result of this physical and chemical mimicry, they have been widely explored as bone substitutes while their bioresorbable properties have even been exploited for therapeutic delivery in cardiac repair (Miragoli et al., 2018; Levingstone et al., 2019). Another example of physical mimicry are mesoporous silicon nanovectors mimicking platelet geometry to bestow NPs with increased circulation properties (van de Ven et al., 2012; Wolfram et al., 2015). Additionally, mesoporous silicon’s highly tailorable degradation parameters allow for the controlled release of a number of loaded payloads, making them favorable for various biomedical applications (Scavo et al., 2015; Yazdi et al., 2015; Fernandez-Moure et al., 2017). Recognizing the superior delivery capabilities of viruses, researchers developed virus-like particles that mimic the capsid structure and virosomes that incorporate the surface glycoproteins into liposome-like NPs (Grgacic and Anderson, 2006; de Jonge et al., 2007). As a result, these NPs have been shown to deliver a wide range of payloads, ranging from antibodies to siRNA to chemotherapeutics (Ashley et al., 2011; Agadjanian et al., 2012; Abraham et al., 2016). Furthermore, the composition of these NPs has included organic materials (e.g., lipids, polymer), metals (e.g., gold), and others (e.g., silica, calcium; Dehaini et al., 2016). Using this combination of physical, chemical and biological biomimicry, researchers were able to demonstrate the promising potential of this strategy to improve upon the previous generations of NPs. While the many variations of this biomimetic approach have been discussed elsewhere (Xia and Jiang, 2008; Yoo et al., 2011; Kwon et al., 2015), this review will focus on a subclass of biomimetic NPs – cell membrane-derived NPs.

In order to improve the ability of these biomimetic NPs to actively engage and communicate with the biological milieu, a subclass of biomimetic NPs centered on the ability to mimic the function and behavior of natural cells has emerged. In particular, these NPs achieve biomimicry by transferring the biological features of native cells, such as red blood cells (RBCs), platelets and leukocytes, onto synthetic NP formulations (Hu et al., 2011; Parodi et al., 2013; Anselmo et al., 2014). In order to synthesize these cell membrane-based NPs, researchers have utilized “top-down” and “bottom-up” approaches. Early work in this field focused on the incorporation of cell-derived ligands, which includes proteins, lipids and carbohydrates (Yurkin and Wang, 2017). As these molecules are key mediators of a cell’s behavior, it was hypothesized that integration of these features onto NPs would endow them with the same behavior. Owing to the development of novel extraction processes, biomimetic NPs now largely use cell membranes derived from native cells. This can be seen in technologies that incorporate the membrane proteins found on leukocytes into a liposomal formulation or the transfer of RBCs *in toto* onto poly(lactic-co-glycolic acid; PLGA) cores (Hu et al., 2011; Molinaro et al., 2016). As a result, these biomimetic technologies express the surface features of native cells and, thereby, mediate the interactions at the nano-bio interface much like a native cell communicates with other cells (Evangelopoulos et al., 2016). With this technology in hand, researchers have explored how these biomimetic NPs can be applied to various diseases.

## Disease Applications

The use of cell membrane-based biomimetic NPs has found therapeutic applications across many diseases, ranging from cancer to cardiovascular disease to infectious diseases. Here we will discuss examples of use of these cell membrane-based biomimetic NPs in these specific disease contexts, placing an emphasis on how these particles communicate with the immune system at the nano-bio interface. In particular, we will demonstrate how these emerging technologies address both sides of the coin of the immune system – a barrier and a target.

### Cancer

Given that a large portion of the work with earlier generations of NPs was done within the context of cancer, this trend continues to remain so with the current generation of biomimetic NPs. Over the years, researchers have explored the use of many cell-membrane coatings (e.g., RBCs, leukocytes, and cancer cells) to achieve targeted delivery to the tumor site (Vijayan et al., 2018; Pasto et al., 2019). As previously described, these membrane-camouflaged NPs face the same challenges posed by the immune system while in circulation. However, the use of native cell membranes has facilitated the interactions at the nano-bio interface such that these NPs remain in circulation and eventually reach the tumor. From the perspective of selective targeting of the tumor, cell membrane-based biomimetic NPs offer the ability to target molecular features of the local inflammation present within the tumor. In addition, the burgeoning interest in the field of cancer immunotherapy has also spurred the search for how these biomimetic NPs can be utilized to modulate the local immune microenvironment in order to induce an anti-tumor response.

**FIGURE 2:**
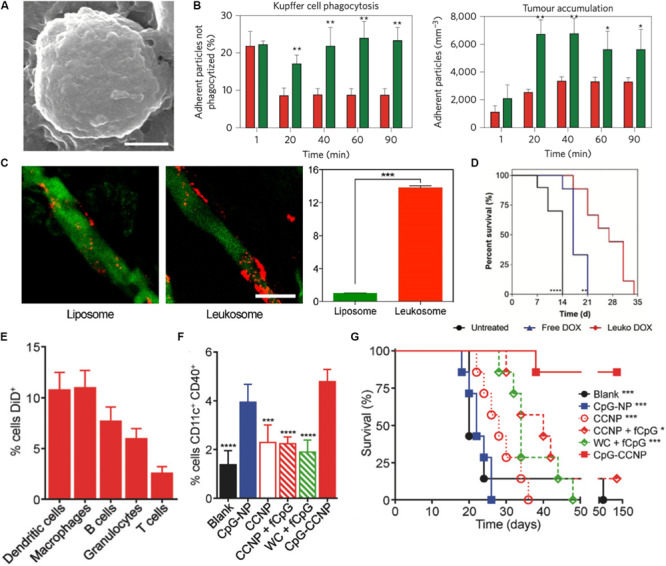
Biomimetic nanoparticles for tumor targeting. **(A,B)** Leukocyte mimicking nanoparticles show reduced uptake by the mononuclear phagocyte system and improved targeting to the tumor. **(A)** SEM image of porous silica nanoparticle cloaked with leukocyte membrane (LLV). Scale bar = 1 μm. **(B)** LLV demonstrated reduced uptake by Kupffer cells (left) and improved targeting to melanoma tumors (right) when compared to bare nanoparticles. **(C)** Leukocyte-based liposomes (Leukosomes) demonstrate greater affinity for inflamed tumor vasculature. Scale bar = 50 μm. **(D)** Improved survival of tumor-bearing mice after loading with treatment with leukosomes loaded with doxorubicin. **(E–G)** Melanoma cell coated nanoparticles encapsulated with CpG (CpG-CCNP) for immunotherapy. **(E)** Uptake of CpG-CCNPs by various immune cells *in vitro* **(F)** *In vivo* dendritic cell maturation following treatment with NPs and other controls **(G)** Overall survival of mice immunized with CpG-CCNPs and other control formulations. Images in **(A,B)** are reproduced with permission from Parodi et al. (2013). Images in **(C)** are reproduced with permission from Martinez et al. (2018). Image in **(D)** reproduced with permission from Molinaro et al. (2020). Images in **(E–G)** reproduced with permission from Kroll et al. (2017).

Tumor targeting cell membrane-based biomimetic NPs have used strategies that mimic many native cell types. RBC-based NPs take advantage of the expression of “don’t eat me’ markers” (i.e., CD47) to improve circulation times and bypass the effects of MPS in order to reach the target tumor (Sun et al., 2019). Along a similar vein, leukocyte membranes have also been utilized to endow NPs with these same properties. Because these biomimetic NPs look much like native immune cells, circulating monocytes do not engage with these NPs and mark them for clearance by the MPS (Parodi et al., 2013; Corbo et al., 2017b; Figures 2A,B). As a result, mimicking the circulation behavior of these cells enables these NPs to have a greater probability of reaching the tumor. In addition to utilizing these natural coatings for evading the immune system while in circulation, researchers have also used these membrane-based NPs to improve the targeting capabilities of NPs to the tumor. For example, liposomes integrated with the membrane proteins of leukocytes showed a 14-fold increase in affinity to inflamed vasculature associated with triple-negative breast cancer tumors when compared to bare liposomes (Martinez et al., 2018; Figure 2C). This improved targeting was found to be attributed to the presence of leukocyte proteins, such as LFA-1 and Mac-1. In fact, blocking these proteins on the NPs significantly reduced their ability to preferentially accumulate within the tumor, highlighting how the presence of these key markers endows these NPs with the ability to behave like leukocytes that inherently target sites of inflammation (Martinez et al., 2018). This leukocyte-based NP was also shown to improve doxorubicin delivery in two models of cancer, melanoma and breast cancer, resulting in a 64 and 142% (respectively) increase in median survival over untreated mice (Molinaro et al., 2020; Figure 2D). Therefore, by mimicking the targeting properties of leukocytes, these NPs were able to more effectively target the tumor and deliver the encapsulated payload. Others have taken advantage of the interactions between platelets and tumor cells to use silica NPs coated with activated platelet membranes to specifically target the circulating tumor cells (CTCs) attributed to metastatic development (Li et al., 2016). In particular, activated platelets and fibrin were found to be physically associated with blood-borne cancer cells. As CTCs travel within the blood and plant the seeds for the development of metastases, the authors hypothesized that use of platelet-mimicking will harness these physical interactions to inhibit metastases. Indeed, this study demonstrated that treatment using these biomimetic NPs resulted in a 40-fold reduction of lung metastases in a triple-negative breast cancer model. In another study, platelet-coated nanovesicles loaded with doxorubicin and functionalized with tumor necrosis factor-related apoptosis inducing ligand (TRAIL) showed greater accumulation to primary breast cancer tumors (Hu Q. et al., 2015). Lastly, studies have also shown the efficacy of using cancer cell membrane-coated NPs (Harris et al., 2019; Wang et al., 2020). For example, 4T1 breast cancer cell membrane coated polymeric NPs displayed longer circulation time and stronger homotypic targeting of primary tumors and metastases (Sun et al., 2016). In this example, the match between protein profile on the NPs and those found on cancer cells enabled the cancer cell to recognize and internalize the NP. These examples highlight how these biomimetic NPs improve upon previous generations by utilizing natural cellular surface features to avoid clearance by immune cells that hinder tumor accumulation and engage directly with the cancer cells upon reaching the site.

Beyond employing these cell membrane-based biomimetic NPs to evade MPS-mediated clearance and selectively target the tumor tissue, researchers have also employed these platforms to induce local changes in the tumor microenvironment. Of particular interest has been the use of these NPs to modulate and tune the immune cell population present both within and in the periphery of the tumor. Work by Xie et al. combined starvation therapy with cancer cell membrane coated NPs to improve the tumor response to anti-PD-1 immunotherapy. Using glucose oxidase loaded mesoporous silica NPs coated with membranes of B16F10 melanoma cancer cells, they were able to not only improve the tumor response to anti-PD-1 therapy, but also induce DC maturity, reduce the percentage of regulatory *T* cells (which support the immunosuppressive environment favorable for tumor growth and survival) and double the effector *T* cell infiltration within the tumor (Xie et al., 2019). Because these NPs utilized cancer cell membranes, the proteomic profile of the NP surface included antigens that can trigger immune cell responses. Upon exposure of these tumor antigens, immature DCs process these antigens and present these peptides to *T* cells (Gardner and Ruffell, 2016). Therefore, these biomimetic NPs presented key molecules that facilitated communication with local immune cells and triggered a subsequent modulation of the *T* cell population within the tumor. Furthermore, the homotypic targeting facilitated by this biomimetic NP enabled selective targeting of the tumor. As a result, delivery of the loaded glucose oxidase significantly reduced glucose metabolism within the tumor cells and, thereby, enhanced tumor cell death. Other studies have also demonstrated the use of cancer nanovesicles to disrupt the PD-1/PD-L1 immune inhibitory axis that serves as the target for clinically approved immune checkpoint inhibitors (Zhang X. et al., 2018). Researchers have also explored the use of biomimetic NPs as cancer vaccines, where the introduction of the NP protects from the development of a tumor when challenged with tumor cells. This was demonstrated in a study utilizing cancer cell membrane coated PLGA NPs as antigen presenting material, which when combined with an immunological adjuvant induced the secretion of pro-inflammatory cytokines by immune cells *in vitro* (Kroll et al., 2017). Using a murine melanoma model, this study demonstrated that these particles were not only taken up by a wide-range of immune cells, but also capable of improving DC maturity and overall survival of the mice by 60% over the course of 5 months (Figures 2E–G). Furthermore, this biomimetic NP platform had strong potency as both a cancer vaccine and treatment regimen for existing tumors.

These studies highlight the use of cell membrane-based biomimetic NPs to not only target the tumor, but also induce changes in the local immune microenvironment. By presenting surface characteristics which immune cells naturally recognize, these cell-like NPs bypass the challenges posed by the immune system, while communicating important signaling cues that facilitate cellular responses vital to mounting an anti-tumor response.

### Cardiovascular Disease

The umbrella of cardiovascular diseases covers a broad spectrum of conditions related to the normal functions of the heart and blood vessels, including myocardial infarction, stroke and high blood pressure (Stewart et al., 2017). From its inception, the pathophysiology of cardiovascular disease is characterized by high levels of inflammation (Golia et al., 2014). However, the underlying cause for many of these conditions is buildup of atherosclerotic plaque (Bobryshev et al., 2016). Under normal conditions, arterial walls resist the accumulation of lipids and macrophages. However, triggers of atherosclerosis, which include hypertension, a diet high in saturated fats and obesity, initiate the expression of adhesion molecules that allow the entry of lipids into the vascular wall and the subsequent recruitment of leukocytes to the affected area (Rafieian-Kopaei et al., 2014). The use of cell membrane-based biomimetic NPs for these applications have focused on mimicking various cell membranes (e.g., as platelets and leukocytes) and protein complexes important for good cardiovascular health [i.e., high-density lipoprotein (HDL); Park et al., 2020].

**FIGURE 3:**
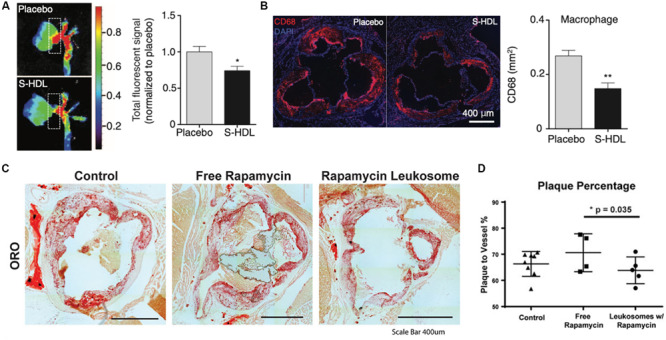
Therapeutic applications of biomimetic nanoparticles in cardiovascular disease. **(A,B)** HDL-mimicking nanoparticles reduce plaque burden and infiltration of macrophages (red) in the aortic roots of atherosclerotic mice. **(C,D)** Rapamycin-loaded leukocyte-based nanoparticles for the treatment of atherosclerosis **(C)** Oil red O staining for lipid deposition in the aortas of atherosclerotic mice treated with or without nanoparticles **(D)** Image quantification of the plaque area with the vessels. Images in **(A,B)** are reproduced with permission from Tang et al. (2015). Images in **(C,D)** are reproduced with permission from Boada et al. (2020).

High-density lipoprotein is a native lipid transporter NP used by the body to transport lipids which possess a natural affinity toward atherosclerotic plaque (Feig et al., 2014). These interactions facilitate the transport of cholesterol away from plaque-laden macrophages to the liver for processing. Therefore, this molecule serves as a model complex whose functions NPs can mimic in order to ameliorate the pathophysiology associated with plaques. In an *in vivo* study with advanced atherosclerotic plaques, researchers were able to inhibit the proliferation of atherosclerotic plaque macrophages through synthesized HDL-mimicking NPs (Tang et al., 2015). This in turn reduced macrophage proliferation by 45% in the aortic roots, reduced expression of inflammatory genes and alleviated atherosclerosis over the course of 8 weeks of treatment (Figures 3A,B). Similar to native HDL, these NPs shifted the movement of cholesterol to the liver and, thereby, prevented proliferation of the macrophages that fuel atherosclerotic plaque. Platelets have also been largely implicated in the progression of cardiovascular disease and demonstrated preferential binding to damaged blood vessels (Kinloughrathbone et al., 1983). Using this behavior as the basis for targeting, platelet-membrane coated NPs have been fabricated using a freeze and thaw process, after which the extracted membranes were fused onto PLGA cores (Hu C. M. J. et al., 2015). These biomimetic NPs not only exhibited increased binding to damaged arteries, but also inhibition of neointima growth (i.e., scar tissue formation) in a rat coronary stenosis model when loaded with docetaxel. Another study utilized platelet derived vesicles to facilitate targeted delivery of cardiac stem cells to sites of injury following a myocardial infarction (Tang et al., 2018). Following treatment with these platelet-modified cardiac stem cells, researchers observed increased cardiomyocyte growth and doubled vessel growth in a rat ischemia model. By combining these two cell sources onto a single NP, they demonstrated how this hybrid particle took on both the targeting features of platelets to damaged blood vessels and the self-renewal properties of stem cells to heal the damaged tissue. In addition to platelets, NPs mimicking RBCs and leukocytes have also been developed for the delivery of therapeutic molecules that aid in the treatment of cardiovascular diseases. For example, RBC-membrane coated dextran polymer NPs loaded with a neuroprotective agent prolonged circulation of this therapeutic molecule while reducing ischemic brain damage in a cerebral artery occlusion model (Lv et al., 2018). Similarly, leukocyte-based NPs loaded with rapamycin also demonstrated preferential accumulation in atherosclerotic plaques in a murine model, curbing local inflammation by reducing macrophage proliferation (Boada et al., 2020). Furthermore, the release of rapamycin from these particles also resulted in reduced plaque burden within the vessels (Figures 3C,D). In this case, the incorporation of the leukocyte proteins onto the NP not only improved targeting to the inflamed site but also induced anti-inflammatory effects that reduced local inflammation at the disease site (Boada et al., 2020).

Taken together, these studies exemplify how cell membrane-based biomimetic NPs can be synthesized to target and treat various aspects of the pathophysiology of cardiovascular diseases. By taking inspiration from the behavior of native cells in this disease context, researchers have implemented novel technologies that mimic the behavior of innate cells while mediating the inflammatory response found across all these disease conditions.

### Infectious Disease

The presence of pathogens in the body due to an infection triggers a multi-faceted immune response aimed at clearing the source itself (Chaplin, 2010). The treatment of the most common infectious diseases, which primarily stem from bacteria, has traditionally relied on the heavy use of antibiotic regimens. With the rise of antibiotic resistance over the years, researchers have developed and tested a host of new technologies that aim to treat infectious diseases while minimizing antibiotic use (Aslam et al., 2018). Cell membrane-based biomimetic NPs have paved the way for a new class of treatments that resolve infections using three key approaches: target the source of infection, neutralize the mechanisms used by pathogens to deactivate natural immune defenses and modulate the immune cells involved in the anti-pathogen response.

**FIGURE 4:**
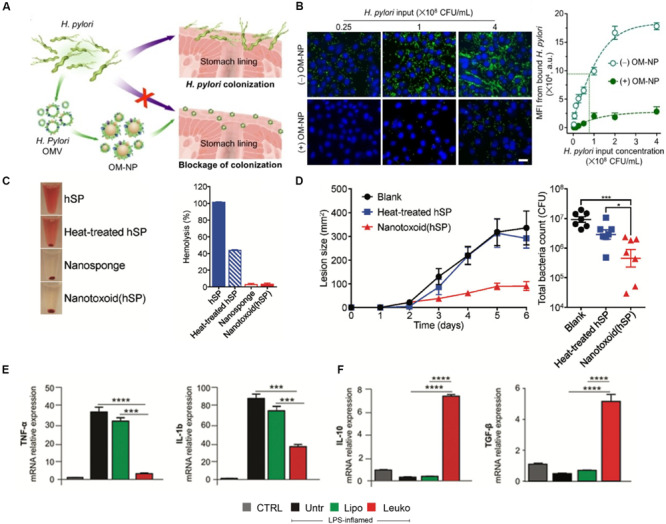
Biomimetic nanoparticles to kill, neutralize and modulate the immune response to pathogens in infectious diseases. **(A,B)** Bacterial membrane coated nanoparticles prevent binding to host cells. **(A)** Schematic representing use of bacterial NPs to prevent *H. Pylori* colonization of stomach tissue. **(B)** Confocal images (left) and quantification (right) of *H. Pylori* (green) adhesion to gastric epithelial cells (blue) with or without treatment with NPs. Scale bar = 25 μm. **(C,D)** RBC-coated nanosponges (Nanotoxoid hSP) as toxoid vaccines against bacterial infections **(C)** Sample images (left) and quantification of hemolysis following treatment with NPs **(D)** Lesion size (left) and total bacterial count (right) in mice following vaccination with NPs and controls **(E,F)** Treatment of sepsis with leukocyte-mimicking liposomes induced reduction of proinflammatory genes **(E)** while increasing expression of anti-inflammatory genes **(F)**. Images in **(A,B)** are reproduced with permission from Zhang et al. (2019). Images in **(C,D)** are reproduced with permission from Wei et al. (2017). Images in **(E,F)** are reproduced with permission from Molinaro et al. (2019).

The first strategy researchers have used to target these infections is the use of cell membrane-based biomimetic NPs that target the pathogen itself. NPs mimicking platelets, epithelial cells and even bacteria themselves have been used to achieve this specificity of targeting. For example, bacteria have been shown to infiltrate platelets and result in platelet aggregation (Fitzgerald et al., 2006). Although platelets play a fundamental role in the host’s defense system, overactivation can lead to the development of hard-to-treat thrombi, creating a niche where the bacteria are protected from the host’s immune system. Capitalizing on this feature of bacteria, researchers designed platelet-coated NPs to effectively deliver antibiotics (Hu C. M. J. et al., 2015). Significant antimicrobial activity was observed in mice that were systemically challenged with a methicillin-resistant strain of bacteria and treated with these biomimetic NPs. Other strategies have used gastric epithelial cell membrane NPs as drug delivery vehicles for antibiotics (Angsantikul et al., 2018). This strategy was particularly unique in that the NPs presented the surface antigens the bacteria would normally recognize on the host’s cells. As a result of recognizing these particular proteins on the NP surface, the bacteria inadvertently internalize these NPs carrying lethal antibiotics. Another approach is to prevent the adhesion of bacteria to the host’s cells by using biomimetic NPs as a competitive binder of target sites (Zhang et al., 2019). This strategy was proven to be effective in a study using polymeric NPs wrapped with bacterial outer membranes of *H. pylori* in order to inhibit the adhesion of this bacteria to the stomach lining (Figures 4A,B). These bacteria mimicking NPs occupied the binding sites normally used by the pathogen to colonize and induce infection. In fact, these biomimetic NPs decreased binding of *H. pylori* to intestinal cells by 6-fold *in vitro* while reducing bacterial colonization in murine stomach tissue by almost 50% *in vivo*. These examples highlight how the surface features of these biomimetic NPs cleverly mediate communication with the target pathogen or prevent its communication with the host cells which ultimately result in the killing of the bacteria itself.

Toxin release is a frequently used mechanisms by which pathogens attack host cells and begin the process of infection (Sastalla et al., 2016). The detection of these deadly toxins is one of the many ways in which the immune system recognizes the presence of these pathogens (Rudkin et al., 2017). However, many of these toxins are also cytotoxic to the immune cells that arrive to clear the pathogen (do Vale et al., 2016). Therefore, cell membrane-based biomimetic NPs have been investigated as toxin neutralizing platforms that can protect immune cells from cell death and enable them to neutralize the pathogen (Fang et al., 2015). Owing to their longer circulation times and ability to interact with the pathogens in circulation, this approach has been shown using primarily RBC-coated NPs. Polymeric NPs were wrapped with the membranes of RBCs and shown to sequester multiples pore-forming toxins (e.g., α-hemolysin, streptolysin-O, and melittin) and protect cells from hemolysis (Hu et al., 2013). Furthermore, these NPs, termed “nanosponges,” did not transfer these toxins to host cell, demonstrating the relative safety of this platform. In a follow-up study, these same nanosponges were shown to act as toxoid vaccines, where the inactivated toxins integrated into the membranes of the NPs protect mice challenged with methicillin-resistant staphylococcus aureus (Wei et al., 2017; Figures 4C,D). The protective properties of these NPs were highlighted by the absence of hemolysis, 4-fold reduction in the lesion size and reduction of the total bacteria count following treatment with the NPs. By capitalizing on the native features of RBCs, such as blood circulation times and the expression of “self-marker” proteins, these biomimetic NPs not only bypass the normal barriers imposed by the immune system, but also protect the very immune cells that often mark them as foreign bodies.

Beyond targeted delivery of antibiotics or neutralization of toxins, cell membrane-based biomimetic NPs have also been shown to modulate the immune response necessary for resolving an infection (Angsantikul et al., 2015). This has been especially demonstrated in models of sepsis, which occurs when infection spreads beyond the local tissue and results in systemic organ dysfunction (Delano and Ward, 2016). Using polymeric cores wrapped in the membranes of macrophages, researchers were able to show the utility of this technology in neutralizing endotoxins that would activate the immune system and sequester proinflammatory cytokines, such as TNF-α and IL-6, in a two-step process (Thamphiwatana et al., 2017). Furthermore, these biomimetic NPs improved the survival of mice challenged with *E. coli* by 60%. These NPs served as decoys for LPS and cytokines by binding these inflammatory factors to the native pattern recognition receptors on the NP surface. By first neutralizing the LPS and then sequestering cytokines, these macrophage-like NPs inhibited a systemic inflammatory response and, thereby, increased survival during septic shock. Another study using macrophage-derived nanovesicles, where the membrane proteins of macrophages are embedded into a liposome formulation, showed similar abilities to reduce the effects of proinflammatory genes, such as TNF-α and IL-1ß, while increasing the expression of anti-inflammatory genes, such as IL-10 and TGF-ß (Molinaro et al., 2019; Figures 4E,F). Although it should be noted that previous studies have shown the ability of bare, synthetic NPs to treat sepsis, these studies have been limited to physicochemical features as a means to interact with the local environment (Casey et al., 2019). In contrast, these cell-derived biomimetic NPs function by mimicking the native signaling patterns of the cell source. As a result, they provide a clear distinction on the mechanism by which the particles impart therapeutic efficacy and, in turn, enable greater control of the NPs interactions with the biological milieu. By modulating key mediators (i.e., cytokines) of the immune response in sepsis, these studies show the ability of these NPs to tune and regulate this delicate balance to prevent overactivation of the immune system, which can lead to septic shock in patients.

### Autoimmune Disease

Autoimmune diseases cover a wide spectrum of diseases, ranging from type 1 diabetes to rheumatoid arthritis to systemic lupus (Theofilopoulos et al., 2017). These diseases are characterized by autoimmunity where the immune system begins to attack the body’s own cells through various means, such as through the production of antibodies against its own cells (Wang et al., 2015). Furthermore, these diseases are characterized by a state of chronic inflammation where the immune system continues to attempt to repair the resulting damage. Although these diseases are currently considered incurable, studies using biomimetic cell membrane-based NPs have shown the emerging potential these technologies possess to intervene and mediate the behavior of the immune system in these disease conditions.

**FIGURE 5:**
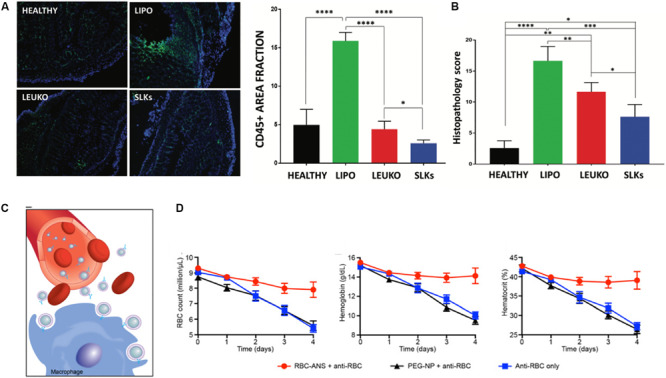
Role of biomimetic nanoparticles in treatment of autoimmune diseases. **(A,B)** Specialized leukocyte-mimicking nanoparticles (SLK) with overexpression of α4β7 integrins for the treatment of inflammatory bowel disease. **(A)** Immunofluorescent imaging for CD45 + immune cells in the colon following treatment with and without nanoparticles resulted in the decreased immune filtration in the SLK group with overall improvement in the histology of the colon **(B)**. **(C,D)** Red blood cell coated nanoparticles (RBC-ANS) for the clearance of pathological antibodies **(C)** Representative schematic demonstrating how RBC-ANS work to neutralize antibodies that would otherwise induce hemolysis **(D)** Significant improvements in RBC count, hemoglobin and hematocount were observed in mice treated with RBC-ANS compared to bare nanoparticles alone. Images in **(A,B)** are reproduced with permission from Corbo et al. (2017a). Images in **(C,D)** are reproduced with permission from Copp et al. (2014).

While the work in this field remains limited, a few studies have shown the therapeutic applications of cell membrane-based biomimetic NPs in diseases such as inflammatory bowel disease (IBD), type II immune hypersensitivity reactions and rheumatoid arthritis. Within these disease conditions, these biomimetic NPs have been shown to act as binding decoys for mechanisms that drive the chronic inflammatory state and serve as mimics of native cells capable of resolving inflammation and repairing tissue damage. Exploiting the mechanisms of *T* cell recruitment during the pathogenesis of IBD, engineered leukocyte membrane mimicking NPs were developed to overexpress α4β7, a key integrin protein on T-lymphocytes, to bind to inflamed mucosal tissue (Berlin et al., 1993). As a result of this overexpression, these “specialized leukosomes” exhibited tighter binding to inflamed endothelia. Furthermore, treatment of DSS-induced IBD mice with these biomimetic NPs resulted in inhibition of edema, reduction of CD45 + immune cells and improved crypt structure (Corbo et al., 2017a; Figures 5A,B). The therapeutic effects observed following treatment with these specialized NPs was speculated to be due to the binding of NPs to receptors that would otherwise be bound by the immune cells that drive this disease. This hypothesis was supported by their observation of lower inflammation levels and reduced immune cell infiltration in the colon of mice treated with the NPs. Therefore, these NPs served as competitive binders for receptors that would otherwise overstimulate the immune response in a negative manner. This approach was also demonstrated with RBC-mimicking NPs for the clearance of pathological antibodies. More specifically, these RBC-based biomimetic NPs acted as binding decoys for antibodies that would otherwise bind to native RBCs and mark them for extravascular hemolysis (Copp et al., 2014; Figure 5C,D). In an induced anemia model, mice treated with these RBC-NPs achieved normal RBC counts and hemoglobin levels. In contrast, mice that did not receive the NPs showed a 60% reduction in RBC count and a 2-fold decrease in hemoglobin levels. Lastly, neutrophil-mimicking NPs have been shown to have significant therapeutic effects in the treatment of rheumatoid arthritis. In this study, NPs were fused with the membranes of neutrophils and tested for their ability to counteract the negative immune response induced during the progression of this disease (Zhang Q. Z. et al., 2018). Similar to the previously discussed examples, these NPs also served as decoys of neutrophil-targeted biological molecules. This is especially important for the treatment of rheumatoid arthritis where reversal of this condition has been linked to reduced neutrophil recruitment to the synovial fluid (Wright et al., 2017). In fact, use of these NPs in two murine models of arthritis resulted in a reduction of joint destruction and suppression of proinflammatory cytokines. These studies demonstrate the versatility biomimetic NPs possess in targeting and tuning the underlying mechanisms that support and drive many autoimmune diseases.

## Conclusion and Perspectives

Significant progress has been made in the modulation of the immune response to NPs, with cell membrane-based biomimetic NPs paving the way for a new generation of innovative therapeutic platforms. Advances in the field of material science and engineering have led to the development of novel synthesis methods capable of transferring the complex surface composition of native cells onto NPs. As we synthesize cell membrane-based biomimetic NPs with specific features, we are directly encoding messages into the surfaces of these particles. Therefore, the messages we encode mediate the communication that occurs between these particles and the biological components they encounter at the nano-bio interface. Researchers must take advantage of these processes to create NPs endowed with fine-tuned properties that can further facilitate the immunomodulatory interactions that are crucial to determining the *in vivo* fate of these delivery systems. The work in this field thus far has demonstrated the wide applications of these biomimetic technologies across many diseases. Not only do these NPs possess the ability to negotiate the barriers imposed by the immune system with ease, their interactions with cells in the surrounding biological environment via the nano-bio interface enable them to directly communicate with the immune cells involved in disease progression. As growing research in immunology and cell biology shed more light on the cell-to-cell interactions that mediate the healing process, researchers can capitalize on this knowledge to develop even more intelligent and intricate cell membrane-based biomimetic NPs.

Despite the many advantages provided by cell membrane-based strategies, a variety of challenges exist that must be overcome for successful translation into the clinic. Chiefly, successful identification and isolation of cell membranes is an extensive, multi-step process and can be limited by the chosen cell source. For example, stem cells can be quite challenging to derive from a patient while other cell sources may demonstrate considerable heterogeneity. In the case of erythrocytes and platelets, while these can be commonly retrieved via transfusion, their lack of a nucleus makes it difficult for gene-based *ex vivo* manipulation. Therefore, in the successful translation of these strategies into the clinic, it is important to consider the source of cells in an effort to obtain relevant quantities of material.

Additionally, as these NPs are derived from native cells, maintaining batch-to-batch consistency is of utmost importance for clinical translation. Current fabrication techniques make it challenging to ensure homogeneous incorporation of integral membrane proteins. While top-down approaches have provided some insight into the development of more controlled structures, additional optimization is still needed. Specifically, as the surface expression of key molecules on these NPs is vital for their behavior *in vivo*, researchers must develop quick screening assays that confirm the incorporation and function of the NPs prior to administration in a patient. Additionally, cell membrane proteins possess a number of features ranging from targeting to cell-cell communication, requiring techniques to selectively isolate desired proteins. Unintended consequences can also arise from the inclusion of undesirable proteins such as the potential to activate the immune system. Furthermore, the incorporation of denatured proteins also raises the risk of immune activation, necessitating methods that allow for identification of essential proteins.

Nevertheless, the growing work in cell-based therapies has spurred the groundwork for formalized standards by which these technologies must adhere. As the standardization of processes and regulations for these technologies grow, it is expected that this can aid in the successful translation of cell membrane-based NPs into the clinic. Furthermore, as the development of novel cell membrane-based NPs continues, it is important to evaluate process development methods that can favor scale up. In all, the work showcased in this review highlights the potential for this technology to be translated from the bench to the bedside.

## Author Contributions

MS and ME wrote the manuscript and compiled the figures. FT and ET aided in the idea conception, development, and manuscript editing process.

## Conflict of Interest

The authors declare that the research was conducted in the absence of any commercial or financial relationships that could be construed as a potential conflict of interest.
